# Effects of Surface Treatment Method Forming New Nano/Micro Hierarchical Structures on Attachment and Proliferation of Osteoblast-like Cells

**DOI:** 10.3390/ma16165717

**Published:** 2023-08-21

**Authors:** Jae-Seung Im, Hyunsuk Choi, Hyun-Wook An, Tae-Yub Kwon, Min-Ho Hong

**Affiliations:** 1 Department of Dental Laboratory Science, College of Health Sciences, Catholic University of Pusan, 57 Oryundae-ro, Geumjeong-gu, Busan 46252, Republic of Korea; 2 Department of Dentistry and Prosthodontics, Daegu Catholic University School of Medicine, 33 Duryugongwon-ro 17-gil, Nam-gu, Daegu 42472, Republic of Korea; hschoi@cu.ac.kr; 3 Department of Dental Science, Graduate School, Kyungpook National University, 2177 Dalgubeol-daero, Jung-gu, Daegu 41940, Republic of Korea; 4 Department of Dental Biomaterials, School of Dentistry and Institute for Biomaterials Research & Development, Kyungpook National University, 2177 Dalgubeol-daero, Jung-gu, Daegu 41940, Republic of Korea; tykwon@knu.ac.kr

**Keywords:** surface treatment, titanium, hierarchical structures, dental implant

## Abstract

Titanium (Ti) and Ti-based alloys are commonly used in dental implants, and surface modifications of dental implants are important for achieving osseointegration (i.e., direct connection between the implant surface and bone). This study investigated the effect of an eco-friendly etching solution—a hydrogen peroxide–sodium bicarbonate mixture—on the surface properties and contact angles of osteoblast adhesion and proliferation on Ti surfaces. Disk-shaped Ti specimens were prepared using different surface treatments (machining, sandblasting, and sandblasting/acid-etching), and they were immersed in the etching solution and ultrasonically cleaned. Surface characterization was performed using scanning electron microscopy, digital microscopy, contact angle analysis, and X-ray photoelectron spectroscopy. MG-63 osteoblasts were cultured on the specimens, and their adhesion to the specimen surface and proliferation were examined using staining and the MTT assay, respectively. Additional etching with the etching solution caused the formation of nano/micro hierarchical structures, increased surface roughness, and enhanced hydrophilicity. Osteoblast adhesion and proliferation were found to improve on the modified surfaces. The eco-friendly etching method has the potential to enhance the biological properties of Ti implant surfaces and thereby improve dental implant performance.

## 1. Introduction

Titanium (Ti) and Ti-based alloys are the most commonly used implant materials in the dental field, owing to their excellent mechanical/physical properties and biocompatibility [1]. When dental implants are used, it is important to achieve osseointegration, which refers to the direct connection between the implant surface and living bone without any soft tissue interference [2]. It is known that the surface topography of implants plays a critical role in the interaction between their surface and adjacent bone tissue [3], and early osseointegration is generally achieved through surface modifications, such as the modification of the chemical composition or surface roughness [4,5]. In particular, rough Ti surfaces have been found to elicit better osteoblast responses than smooth ones [6]. Protein/implant and cell/implant interactions are also influenced by the surface morphology of the implant [7].

Structures such as scallops, bulges, and holes that are similar in size to cells can significantly affect osseointegration. The response of cells to microscale surface features, which include changes in their shape, location, and polarization, is known as contact induction [8]. It is widely acknowledged that different levels of surface roughness lead to different cellular responses; for example, micro-rough structures are favorable for cell attachment, while nano-rough structures promote cell differentiation, protein synthesis, and gene expression [9,10]. Moreover, nanoscale surface features have been shown to enhance antimicrobial properties [11,12], and nanomaterial-based antibacterial photodynamic therapy inhibits bacterial-plaque-induced oral diseases [13]. Novel physiologically active and therapeutic dental polymer materials that inhibit periodontal pathogens and biofilms can also suppress periodontitis and protect tooth structure [14] and have high therapeutic potential for reducing the risk of inflammation around implants. In particular, rough surfaces can significantly enhance mechanical interlocking between the implant material and bone tissue, resulting in high stability and strong fixation of the implant.

In the medical field, Ti and its alloys have a lower osteoblast attachment than modified surfaces because of their machined surfaces. In a previous study, faster osteoblast attachment was observed on a modified surface than on smooth, machined, or polished surfaces [15]. The bonding between bone and metal can be strengthened through surface modification, which can be performed using methods such as resorbable blasting media (RBM), sandblasted large-grit acid-etching (SLA), chemical vapor deposition (CVD), plasma spraying, and plasma electrolytic oxidation (PEO) [16]. The surfaces of Ti and Ti alloy implants are commonly modified through SLA, a process that involves blasting the surface with coarse abrasive particles and then subjecting it to dual acid etching using strong acids [17]. This process produces an isotropic topography with irregularities on the macroscale and interconnected cavities on the micron and sub-micron scale. The enhanced osseointegration properties of the surfaces are believed to result from stronger mechanical interlocking with the surrounding bone, as well as increased surface area, surface energy, protein adsorption, and cell adhesion during the initial stages of wound healing [18,19,20]. Compared with machined implants, Ti surfaces with micro-roughness have been observed to cause variations in the proliferation, differentiation, and secretion patterns of osteogenic cells [21,22,23].

Wennerberg and Albrektsson found that high surface roughness accelerates bone formation [24]. According to studies conducted by Berglundh et al., an implant’s surface should be moderately rough. Hydrophilicity also plays a significant role in implant performance [25]. To enhance an implant surface’s hydrophilicity and bioactivity, methods such as physical, chemical, and biological modifications have been employed [26,27]. Sandblasted and acid-etched surfaces show good implant–cell interactions, which makes them a preferred choice for most dental implants used clinically [28]. The modification of implant surfaces not only enhances bone healing but also improves the primary stability of the implant–bone interface. However, high surface roughness can also increase plaque accumulation [29,30]. Therefore, there is a need for effective implant decontamination strategies that do not involve the alteration of the surface topography to ensure the long-term stability of surface-treated dental implants, especially in patients with compromised conditions [29,30,31].

Among the different surface modification methods, SLA has been the most commercially successful in the field of Ti-based dental implants [32,33]. The presence of micron-sized surface structures facilitates robust mechanical interlocking between the implant and the surrounding bone, resulting in a significantly larger contact area for stable implant fixation [34]. In this method, sandblasted Ti implants with micron and submicron topographies are realized by immersing the implants in an etching solution consisting of concentrated sulfuric acid (H_2_SO_4_) and hydrochloric acid (HCl) [3]. However, in the case of Ti-based alloys subjected to SLA, poor osteoblast adhesion in the early stages of the placement of the alloys poses a major problem [32,35]. Furthermore, this technique involves the use of strong acids and heat; hence, it requires long and complex post-etching cleaning processes [3,36].

Recently, an eco-friendly Ti implant surface modification technique was developed. In this technique, a hydrogen peroxide (H_2_O_2_)/sodium bicarbonate (NaHCO_3_) mixture is used as the immersing solution for Ti etching, instead of strong acids, such as H_2_SO_4_ and HCl [3,36]. H_2_O_2_/NaHCO_3_ solution has the advantage of being eco-friendly, and it can prevent the corrosion of alloys; moreover, in the solution, H_2_O_2_ is not decomposed by NaHCO_3_ [37,38]. Simple immersion in the oxidative solution produces reproducible nano/micro structures on Ti implant surfaces without any need for sandblasting [3]. This new technique may be applied to Ti implants subjected to SLA to further enhance the biological properties of their surfaces.

This study tested the null hypothesis that etching with an eco-friendly solution is ineffective. To validate this hypothesis, we analyzed the surface morphology and 3D profile images of specimens, which were obtained using scanning electron microscopy, to determine the effect of additional etching of Ti surfaces subjected to SLA and subsequent treatment (machining or sandblasting) with an H_2_O_2_/NaHCO_3_ mixture on the Ti surface properties. Contact angles and osteoblast adhesion and proliferation on the surfaces were also evaluated.

## 2. Materials and Methods

### 2.1. Sample Preparation

A total of 60 disk-shaped (diameter: 10 mm; thickness: 3 mm) grade 4 Ti specimens (MEGAGEN Implant Co., Ltd., Daegu, Republic of Korea) were prepared, and they were divided into three groups based on their processing: machined (M), sandblasted (SL), and sandblasted/acid-etched groups. The M group specimens were not subjected to any surface treatment, the SL group specimens were sandblasted with Al_2_O_3_ grit with a size of 0.25–0.50 m [39], and the SLA group specimens were sandblasted with Al_2_O_3_ grit with a size of 0.25–0.50 m and subjected to acid-etching and subsequent etching with HCl (10–16%)/H_2_SO_4_ (68–75%) at a temperature of 80–90 °C [39]. Half of the specimens (10 specimens) in each of the three groups were immersed for 2 h in a 30 wt% H_2_O_2_/5 wt% NaHCO_3_ aqueous mixture at room temperature [3] and classified into new groups (ModM, ModSL, ModSLA) (see Table 1). The different groups of specimens used in this study are listed in Table 1. The etched specimens were ultrasonically cleaned for 15 min in each of three solvents, namely acetone, ethanol, and water, and then air-dried.

### 2.2. Surface Characterization

Field emission scanning electron microscopy (FE-SEM, SU8010, Hitachi, Tokyo, Japan) was used to analyze the surface morphology of the unetched and etched Ti specimens. Specimen morphology was imaged at magnifications of 1000×, 5000×, and 50,000× in the secondary electron mode under high vacuum conditions at an acceleration voltage of 15 kV.

Surface roughness data and 3D images of the specimens were obtained using a digital microscope (VHX-7000, Keyence, Itasca, IL, USA), and the roughness data were analyzed with VHX-H5M software (Keyence, Itasca, IL, USA). The specimen was positioned at a tilt angle of 0° to obtain images with a magnification of 300×. A total of 20 images of size 256 × 256 μm^2^ were captured and aligned to obtain surface roughness data and 3D images (*n* = 3) [40]. Three specimens from each group were evaluated by observing ten random spots on each of them, and the average Ra (mean surface profile roughness) and surface texture scan Sa (the center plane average) values were calculated.

The hydrophilicities of the nano-, micro-, and hierarchical micro/nano-structured surfaces were evaluated using the sessile drop method with a contact angle analyzer (Phoenix-MT(A), SEO Co., Ltd., Suwon-si, Republic of Korea). At room temperature, droplets of equal volume (1.0 μL) were dispensed onto the specimen, and the left and right angles were measured (*n* = 3). The contact angle was analyzed using an image analysis program, Surfaceware 7 software (SEO Co., Ltd., Suwon-si, Republic of Korea). The surface chemistry was investigated using X-ray photoelectron spectroscopy (XPS, Nexsa, Thermofisher, Oxford, UK) with Al Kα radiation. The binding energies for each spectrum were calibrated on the basis of the C 1s spectra at 285.0 eV.

### 2.3. Cell Culture and Adhesion

The test operation was performed on a clean bench under strict aseptic conditions, and the test samples were subjected to ultraviolet (UV) sterilization for 30 min [41]. The samples were placed in a 24-well plate, and a cell suspension was seeded on them. The plate was then incubated at 37 °C with 5% CO_2_ for different durations (1 and 24 h) for culturing. After culturing, the samples were washed twice with phosphate-buffered saline (PBS) and fixed with 2% glutaraldehyde at room temperature for 30 min. Subsequently, they were dehydrated (by increasing the concentration of ethanol), dried, immersed in tetramethylsilane for 10 min, and air-dried at room temperature for 1 or 24 h. The dried samples were platinum-coated and observed under a scanning electron microscope (FE-SEM, SU8010, Hitachi, Tokyo, Japan) at an acceleration voltage of 10 kV and at 2000× magnification.

The morphology of the cells grown on each specimen was determined by staining them. MG-63 human osteoblasts were the cells grown, and 1 × 10^5^ cells were aliquoted and cultured [42]. The cultured cells were stained with green fluorescent Alexa Fluor™ 488 phalloidin (Lot No. M18I049, Thermo Fisher Scientific, Bend, OR, USA) and washed with phosphate-buffered saline. Each specimen was stained with ProLong™ Gold Antifade Reagent with DAPI (Lot No. 2305156, Thermo Fisher Scientific, Bend, OR, USA) for microscopic observation, mounted on a microscope slide, and observed under a fluorescence microscope (BX43, Olympus, Tokyo, Japan) at 400× magnification [43].

### 2.4. Cell Proliferation

Cell proliferation on the Ti surfaces was evaluated using a modified MTT (3-[4,5-dimethylthiazol-2-yl]-2,5 diphenyl tetrazolium bromide) assay [39], which was performed according to Annex C of ISO 10993-5 through direct contact [44]. The test operation was carried out on a clean bench under strict aseptic conditions, and the test samples were prepared using UV sterilization for 30 min [41]. Before the test, the cell monolayer culture and its morphology were examined under a microscope. In order to directly expose the test sample, we added portions of the test sample to each well of a 24-well plate, which served as a culture container. Subsequently, a specific amount of cells (4 × 10^4^ cells/well) was evenly pipetted into each well. In the case of the control group, the cell suspension (4 × 10^4^ cells/well) was dispensed into another culture vessel and cultured and incubated at 37 °C in a 5% CO_2_ incubator. After 24 h, the existing culture medium was removed, and a new culture medium was added to the control group. Fresh serum medium was used as the negative control, and a serum medium supplemented with 10% dimethyl sulfoxide (DMSO) was used as the positive control. The incubation duration was 24 h. After culturing the test group and control group for 24 h, we observed and photographed cell growth inhibition and lysis under a microscope. After imaging of the cultured cell suspension, the existing culture medium was removed, MTT solution was added, and the cell suspension was further incubated at 37 °C in a 5% CO_2_ incubator for 2 h. The MTT solution was then removed. DMSO was added to each well, and the plate was shaken. The DMSO amount added to the different wells was identical. After the test sample was removed from the incubator, the absorbance was measured at 570 nm with a microplate reader (ELISA analyzer Sunrise, Tecan Trading AG, Männedorf, Switzerland).

### 2.5. Statistical Analysis

For the surface roughness, contact angle, and cell viability results, Student’s *t* test was used to assess the significance of differences between the pre-etched surface and the additionally etched surface (*α* = 0.05).

## 3. Results and Discussion

**Figure 1:**
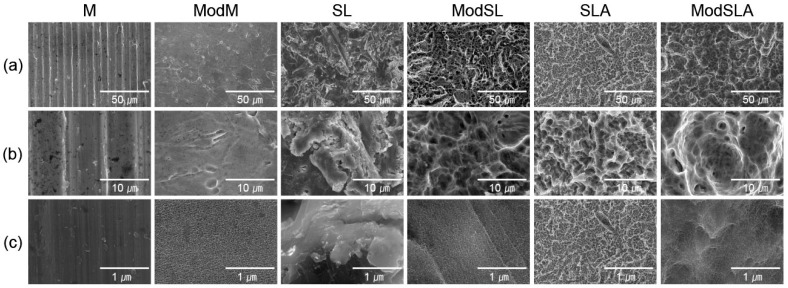
Surface morphology of the Ti alloys used in this study: (**a**) 1000× magnification, (**b**) 5000× magnification, and (**c**) 50,000× magnification. Scale bars are (**a**) 50, (**b**) 10, and (**c**) 1 μm. M: machined surface; ModM: machined surface + eco-friendly solution etching; SL: sandblasted surface; ModSL: sandblasted surface + eco-friendly solution etching; SLA: sandblasted/acid-etched surface; ModSLA: sandblasted/acid-etched surface + eco-friendly solution etching.

Figure 1 shows scanning electron microscopy (SEM) images of all groups listed in Table 1. For the M group machined with cutting tools, typical groove pattern images and profiles were obtained, whereas the SL group had a sharp, fractured surface since the sandblasting particles were sprayed onto the surface of milled Ti. The SLA groups showed larger and deeper cavities, which resulted from sandblasting, than the M groups, and small micropores caused by acid-etching [45]. For the machined Ti surfaces that were etched, low-magnification images (1000× and 5000×) showed the microtopography of the surfaces, and high-magnification (50,000×) images clearly revealed the formation of nanostructures on the surfaces [3]. In the additionally etched ModM, ModSL, and ModSLA groups, in addition to cavities and microstructures similar to those found on the SLA surfaces, nanochannels with a comb-like pattern were newly formed [2].

**Figure 2:**
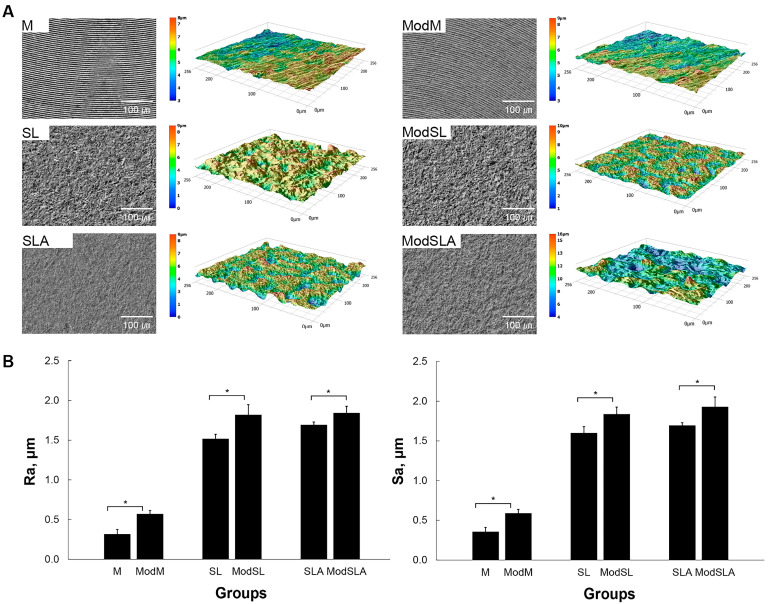
Three-dimensional profile and quantitative topographical evaluations of Ti surfaces. (**A**) Three-dimensional scanning images constructed from digital microscope images; (**B**) results of profile analysis in which Ra (average roughness of profile) and Sa (the center plane average) were evaluated. M: machined surface; ModM: machined surface + eco-friendly solution etching; SL: sandblasted surface; ModSL: sandblasted surface + eco-friendly solution etching; SLA: sandblasted/acid-etched surface; ModSLA: sandblasted/acid-etched surface + eco-friendly solution etching (* *p* < 0.05).

**Figure 3:**
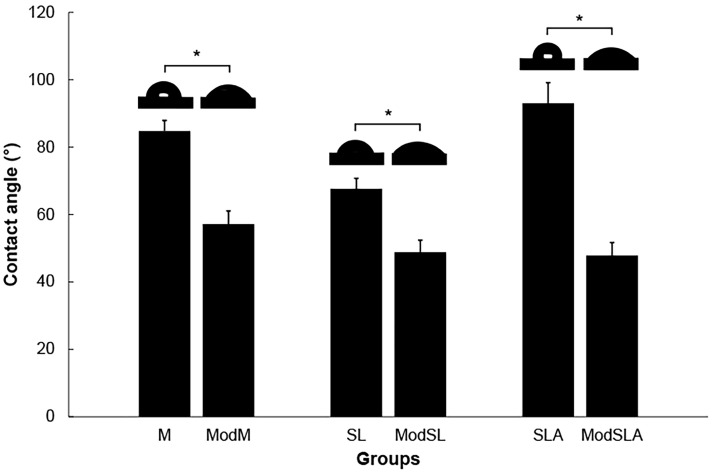
Contact angle graphs and images of the Ti surfaces. M: machined surface; ModM: machined surface + eco-friendly solution etching; SL: sandblasted surface; ModSL: sandblasted surface + eco-friendly solution etching; SLA: sandblasted/acid-etched surface; ModSLA: sandblasted/acid-etched surface + eco-friendly solution etching (* *p* < 0.05).

Figure 2A is a side-by-side indication of SEM images of all Ti surfaces and 3D profile images generated from the corresponding SEM images. Figure 2B shows the surface roughness values (Ra and Sa). Figure 3 shows the water contact angles of all Ti specimens. The additional etching significantly increased the Ra and Sa values in all cases (*p* < 0.05) because of the formation of nano/micro hierarchical structures on the surfaces (Figure 2A,B), but significantly decreased the contact angles (*p* < 0.05) (Figure 3). Before additional etching, the SLA group showed higher contact angles than the M group. For specimens that were not etched with the eco-friendly solution, the Ti surface was hydrophobic. However, in the case of the groups etched with the eco-friendly solution, the Ti surface became hydrophilic. Furthermore, in these groups, the higher wettability of Ti surfaces treated with the H_2_O_2_/NaHCO_3_ mixture was directly associated with the unique nanotopography of interconnected comb-like nanochannels [36]. Kapil et al. [46] stated that hydrophobic surfaces have a high contact angle, low adhesion, low wettability to water, and low surface free energy. In contrast, hydrophilic surfaces have a low contact angle, high adhesion, high wettability to water, and high surface free energy. The main objective of preparing a superhydrophobic surface is to obtain a nanostructured surface through chemical composition changes to increase the water surface tension on the contact surface. This was because surface wettability was highly dependent on surface energy. High surface wettability improves the interaction between the implant surface and the biological environment and enhances cellular activity [47].

MacDonald et al. [48] and Rupp et al. [49] reported that osseointegration is easily achieved when the wettability of an implant is excellent. An implant reacts with the surrounding tissue fluids in the early stages after its placement, and the adsorption of cell adhesion proteins, such as fibronectin, occurs on its surface. In particular, implants with rough surfaces and high surface energies show high protein adsorption in the initial stages.

Ti-based implants with high surface roughness and a large surface area show high bioactivity. Furthermore, the mechanical stability between the bone and the implant is high after the implant’s placement [50]. In particular, a high surface energy results in a surface morphology that can effectively retain blood clots [51]. Boyan et al. [52] reported that surface roughness influences cell behavior, with rough surfaces promoting the adhesion and proliferation of osteoblastic cells because of high collagen synthesis, and smooth surfaces being more favorable for the attachment of fibroblast and epithelial cells.

Junker et al. [53] defined surface roughness in the range of 1–10 μm as micro-roughness and reported that micro-roughness maximizes the interlocking between the implant surface and mineralized bone. Brett et al. [35] reported that nanometer roughness in the range of 1–100 nm plays an important role in protein adsorption and osteointegration involving osteoblastic cell attachment. In this study, the additionally etched groups showed micro-roughness and comb-like nano/micro-roughness. A moderately rough surface (Sa: 1.0–2.0 μm) has been reported to enhance osteoblast adhesion to Ti implants.

Storing cleaned Ti implants in water to maintain the surface free energy of the TiO_2_ surface layer can render the implant surfaces chemically active [54]. By contrast, air exposure can immediately reduce the wettability of a clean TiO_2_ layer, owing to the spontaneous adsorption of hydrocarbons and carbon dioxide [54]. The contact angles of the additionally etched SLA surfaces were found to be lower than those of the etched machined surfaces. To minimize the initial hydrophobicity of SLA surfaces caused by microtopography and atmospheric contamination, studies have proposed the use of SLActive surfaces and normal saline as the storage medium. However, there is no strong evidence showing that SLActive is superior to SLA surfaces in immediate and/or early occlusal loading protocols [30].

**Figure 4:**
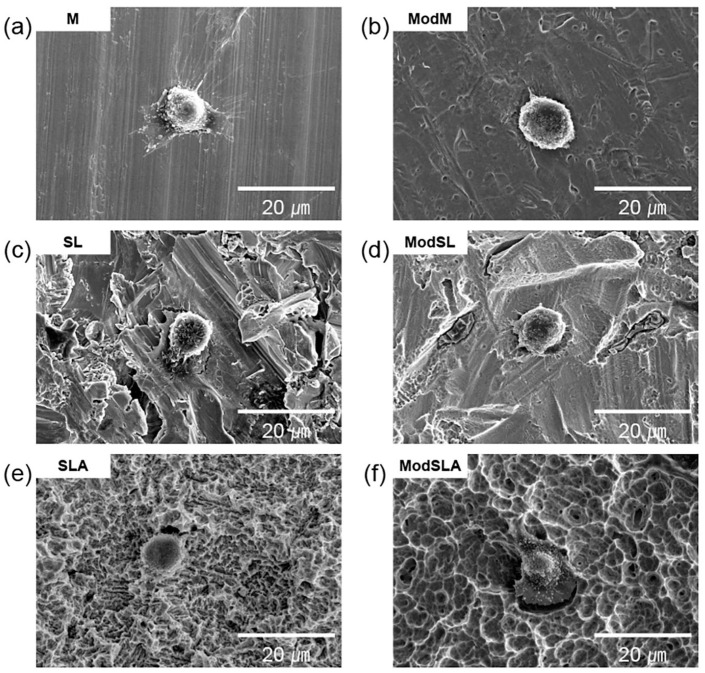
Typical SEM images showing adhesion of osteoblasts cultured for 1 h on grade 4 Ti surfaces at 2000× magnification: (**a**) machined surface, (**b**) machined surface + eco-friendly solution etching, (**c**) sandblasted surface, (**d**) sandblasted surface + eco-friendly solution etching, (**e**) sandblasted/acid-etched surface, and (**f**) sandblasted/acid-etched surface + eco-friendly solution etching.

**Figure 5:**
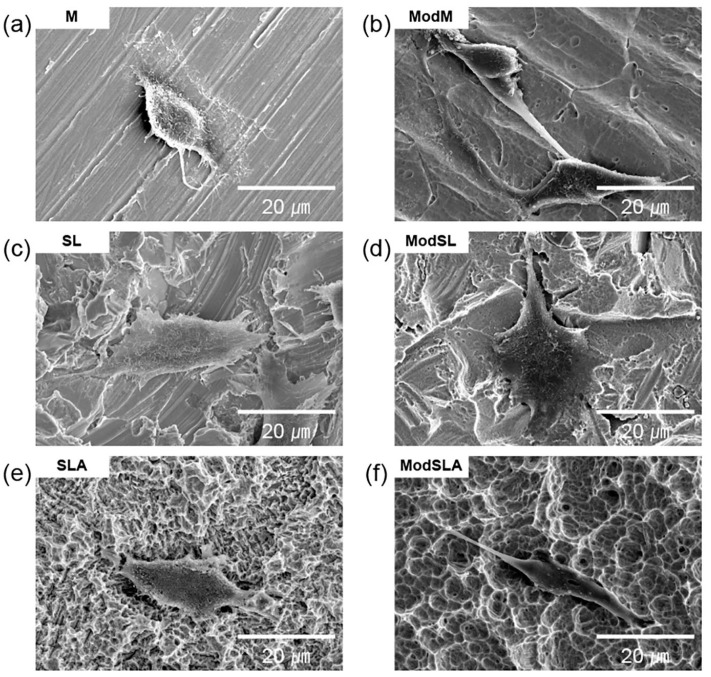
Typical SEM images showing adhesion of osteoblasts cultured for 24 h on grade 4 Ti surfaces at 2000× magnification: (**a**) machined surface, (**b**) machined surface + eco-friendly solution etching, (**c**) sandblasted surface, (**d**) sandblasted surface + eco-friendly solution etching, (**e**) sandblasted/acid-etched surface, and (**f**) sandblasted/acid-etched surface + eco-friendly solution etching.

Figure 4 and Figure 5 depict SEM images showing the morphology of cells cultured on sample surfaces for 1 and 24 h, respectively; the images are shown at 2000× magnification. After 1 h of culture, the cells in every group were similar and circular, and the number of cells was negligible. On the other hand, after 24 h of culture, the cells were spread more uniformly on the entire surface than those cultured for 1 h. Furthermore, the morphology of osteoblasts showed that they were better spread on the additionally etched specimens compared with the cells on the unetched specimens. In particular, the ModSL sample showed a better maintained comb-like microstructure and surface micro-roughness than the ModSLA sample. This shows that treatment with the eco-friendly solution after sandblasting resulted in a superior surface compared to SLA treatment. Previous studies have identified factors contributing to the attachment and proliferation of osteoblasts. Kilpadi et al. [55] reported that the passivation process performed with 20–45% nitric acid according to the American Society for Testing and Materials (ASTM) F86 protocol can minimize the corrosion of Ti. Furthermore, the cell attachment mechanism can be expected to improve when surface energy is increased. Pan et al. [56] reported that 30% peroxide treatment increased the thickness of the TiO_2_ layer on a Ti surface. Ti implant surface reacted with Ca/P in body fluids to form a hydroxycarbonated apatite (HCA) layer that promoted mineralization.

**Figure 6:**
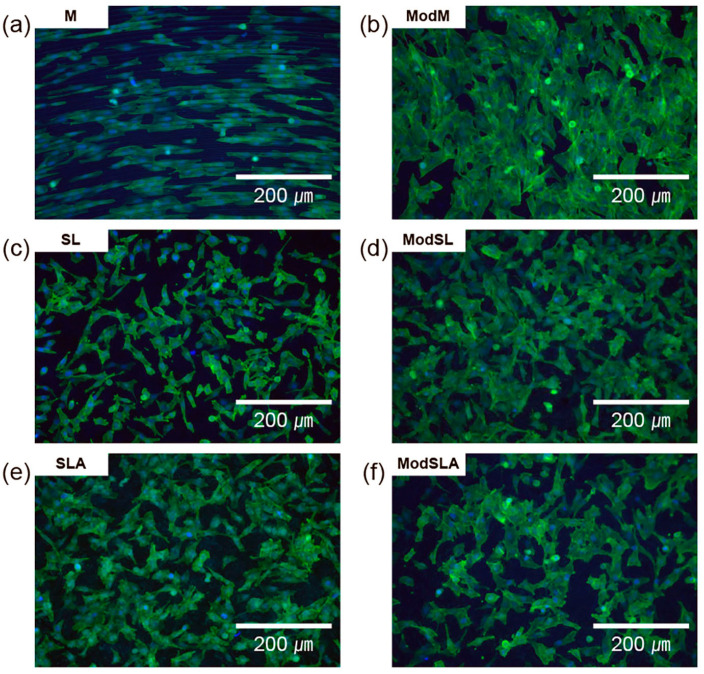
Fluorescence images of LIVE/DEAD staining of MG-63 cells that were cultured on grade 4 Ti surfaces at 200× magnification: (**a**) machined surface, (**b**) machined surface + eco-friendly solution etching, (**c**) sandblasted surface, (**d**) sandblasted surface + eco-friendly solution etching, (**e**) sandblasted/acid-etched surface, and (**f**) sandblasted/acid-etched surface + eco-friendly solution etching.

Figure 6 shows the results of cell staining before and after etching. Cell shapes were similar in the SL and SLA groups, except for the surface of the M group, before etching. However, after etching, the surfaces of all groups had better cell shapes, and similar to the results of cell adhesion, the surface adhesion after etching was higher than that before etching.

Implant surface treatments have been found to impact bone formation and bone remodeling, and several studies have reported that the roughness of an implant surface has a positive effect on osteoblast activity [57]. Furthermore, through cell response experiments involving osteoblasts, it has been reported that implants with irregular rough surfaces exhibit high cell attachment [31,58].

However, only a limited number of studies have directly compared sandblasted surfaces with sandblasted and etched surfaces [25]. Several studies that have investigated osteoblast differentiation associated with high surface micro-roughness appear to have focused on machined or polished Ti surfaces. They considered different micro-roughness levels of surfaces of different groups (such as the machined group and sandblasted group) subjected to various surface treatments [59]. On the other hand, studies that have directly compared the effect of etched surfaces with that of sandblasted and etched surfaces on osteoblast behavior have found higher osteoblast differentiation on etched surfaces [60].

**Figure 7:**
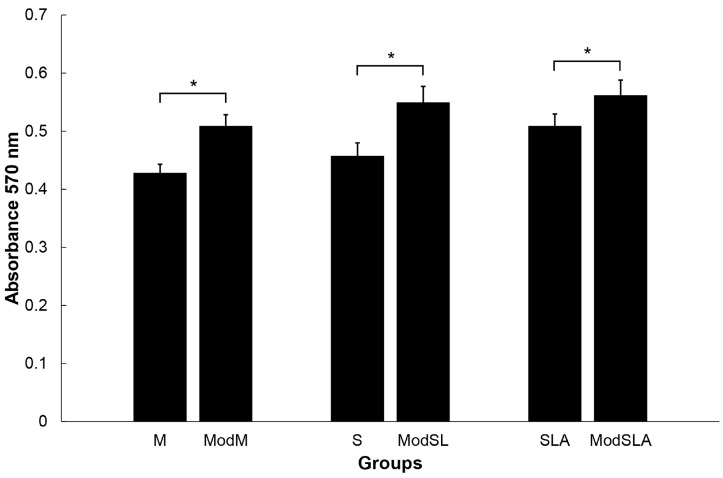
Viability of osteoblastic cells cultured on Ti surfaces on day 1 (* *p* < 0.05).

Figure 7 shows the cell viability results, expressed by the optical density at 570 nm, for all Ti specimens. On day 1, the additional etching did not show any increased cell survival results compared with the unetched conditions (*p* > 0.05). These findings indicate that the additional etching and consequently the formation of nano/micro hierarchical structures on the Ti surfaces (SLA as well as machined) definitely enhanced human osteoblast proliferation.

These results agree with the results of Conserva et al. [61], who found higher differentiation after additional eco-friendly solution etching compared with SLA surfaces. Studies have also investigated the effect of implant surface properties on cell attachment and proliferation. Rosalez-Leal et al. [62] and Keller et al. [60] observed higher attachment of cells on an SLA surface after one hour. However, when compared with a surface etched with an eco-friendly solution, higher proliferation was observed after 24 h. Except for the study of Keller et al., who evaluated osteoblast attachment at a single time point (1 h), our findings corroborate the results of previous studies [60].

**Figure 8:**
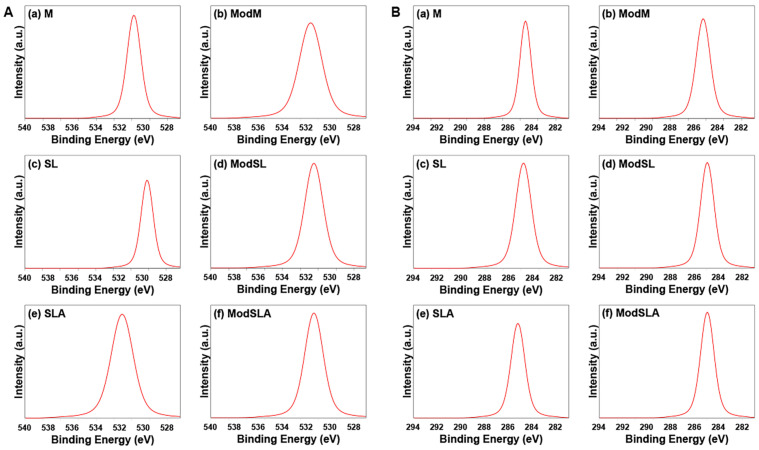
O 1s spectra (**A**) and C 1s spectra (**B**) of all types of Ti surfaces. (**a**) Machined surface, (**b**) machined surface + eco-friendly solution etching, (**c**) sandblasted surface, (**d**) sandblasted surface + eco-friendly solution etching, (**e**) sandblasted/acid-etched surface, and (**f**) sandblasted/acid-etched surface + eco-friendly solution etching.

**Figure 9:**
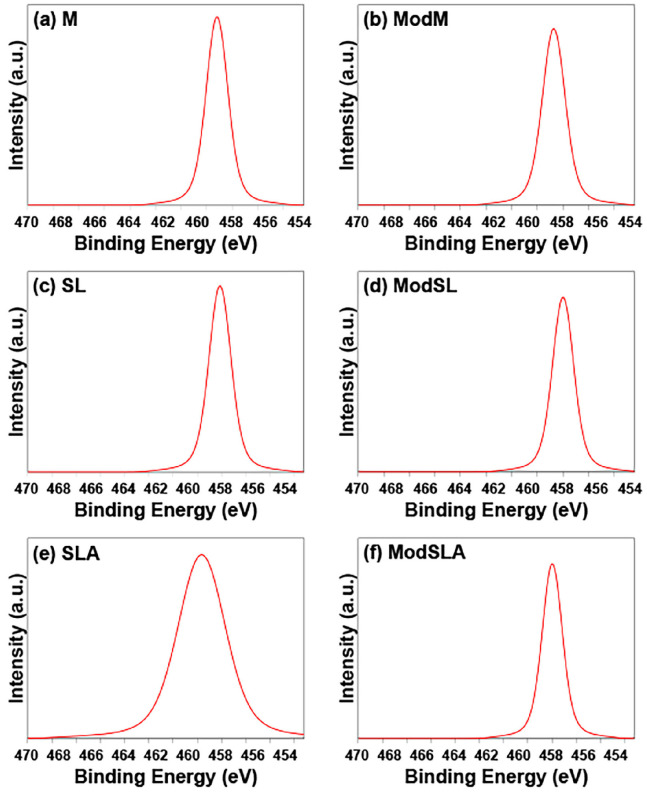
Ti 2P spectra of all types of Ti surfaces. (**a**) Machined surface, (**b**) machined surface + eco-friendly solution etching, (**c**) sandblasted surface, (**d**) sandblasted surface + eco-friendly solution etching, (**e**) sandblasted/acid-etched surface, and (**f**) sandblasted/acid-etched surface + eco-friendly solution etching.

The binding energies of Ti 2p, O 1s, and C 1s core levels are shown in Figure 8 and Figure 9. The figures show a comparison of the intensities of the different elements. The O 1s peak of TiO_2_ was observed around 530 eV for all specimens, and the Ti 2p peak was observed around 458 eV, with a sub-peak around 464 eV. The C 1s peak, supposed to originate from a hydrocarbon (C-H), was observed around 285 eV, with a sub-peak that was attributed to a carbonyl group being observed around 288 eV. Kang et al. [63] noted that the standard binding energies of Ti implant surfaces were as follows: Ti 2p: 458.7 eV; O 1s: 530.1 eV; and C 1s: 284.8 eV. They also observed that when an additional cleaning treatment was performed, the Ti 2p peak split into Ti 2p1 and Ti 2p3 peaks. In other words, the Ti 2p peak was separated into Ti 2p1 and Ti 2p3 peaks at 458.7 ± 0.3 eV for TiO_2_, 457.1 ± 0.3 eV for Ti_2_O_3_, and 455.3 ± 0.1 eV for TiO. Therefore, the binding energy of the Ti 2p peak measured in the current study ranged from 458.4 to 459.2 eV, indicating the formation of a TiO_2_ oxide layer.

Table 2 shows the relative atomic concentrations (at%) and the binding energy of the surface residual elements in the specimens subjected to different surface treatments. The amount of O was the largest in the SL group, probably because of the absorption of O from the air during the sandblasting treatment, followed by the ModSL group. The amount of residual C was the largest in the M group and in the unetched specimens, and it was smallest in the SL and Mod SL groups. The main peaks were Ti and O, while the weak peak was C and resulted from carbon contamination. These observations were consistent with the results of XPS analysis of the surfaces of all specimens.

The residual amount of C in the ModSL group etched with the eco-friendly solution after sandblasting was lower than that in the M and SLA groups, while the residual amount of O was higher. Therefore, the production of TiO_2_ was higher in the ModSL group, which would have increased the attachment area and the speed of osteoblast proliferation. Consequently, the rate of osseointegration was increased because of the migration and proliferation of osteoblasts, and when an implant treated with eco-friendly solution etching after sandblasting was implanted, its initial stability improved, and the interfacial contact surface with bone tissue increased. This resulted in the removal torque value increasing to guarantee the long-term success rate of the implant. The combination of sandblasting treatment and eco-friendly etching treatment has the potential to replace the existing SLA treatment method involving a strong acid mixture (HCl/H_2_SO_4_).

When the Ti specimen was treated with the eco-friendly H_2_O_2_/NaHCO_3_ mixture, it exhibited a nanoscale surface morphology with a comb-like pattern, and the surface roughness and wettability increased. Previous studies, including those of Kim et al. [3], have suggested that the removal effects of Ti surface residues could be expected from the treatment of a Ti alloy specimen with an eco-friendly H_2_O_2_/NaHCO_3_ mixture. In other words, a surface cleaning effect without any change in the surface chemical composition was observed, as H_2_O_2_ is easily decomposed into H_2_O and O with the aid of NaHCO_3_. Moreover, H_2_O_2_ in the eco-friendly mixture caused the formation of a hierarchical structure in which micro-pits and comb-like nano-channels were formed. Furthermore, the formation of the hydroxyl radical (OH), a strong oxidizer, resulted in the Ti surface being oxidized, which increased cell affinity, wettability, and hydrophilicity [36].

The mechanism underlying the formation of nano/micro-textures on a Ti surface using the H_2_O_2_/NaHCO_3_ mixture is not yet fully understood. It is known that NaHCO_3_ is a slightly alkaline powder with a pH of around 8, and H_2_O_2_ is a strong oxidizer. When NaHCO_3_ is added to a 30% H_2_O_2_ solution with a stabilizer, the pH of the resultant changes significantly from 1.9 to 7.8. It is believed that when a Ti disk is submerged in this mildly alkaline mixture, H_2_O_2_ reacts with Ti ions, especially Ti^3+^, to generate hydroxyl radicals, perhydroxyl radicals, and superoxide anion radicals. Among these reaction products, the hydroxyl radical is the most potent oxidizer and is likely responsible for the observed surface oxidation. Regardless of the cause of oxidation, this innovative oxidative solution shows promise as an alternative to highly concentrated acid solutions for use in Ti surface modification [36].

This study examined whether the acid-etching process, which appears to be problematic in the commonly used surface treatment process, can be replaced with an eco-friendly solution by comparing the ModSL specimen (etched with the eco-friendly H_2_O_2_/NaHCO_3_ mixture after sandblasting) with the ModSLA specimen (subjected to SLA and etched with HCl/H_2_SO_4_, a commonly used strong acid mixture). It was found that the biological surface characteristics of the former were somewhat better than those of the latter. Therefore, the eco-friendly H_2_O_2_/NaHCO_3_ mixture has the potential to replace the currently used HCl/H_2_SO_4_.

The limitations of our study include the evaluation of surface energy changes through a method that has limitations called the contact angle method. This method, although commonly used, may not fully capture the complexity of surface interactions. Furthermore, the analysis of cell behavior was limited to single cells, whose behavior may not be similar to the collective behavior of multiple cells in a biological context. Additionally, there were experimental constraints in our study. For instance, cell viability experiments were conducted for a relatively short duration of 24 h, which might not be sufficient for the capture of long-term effects. Another limitation is that the study focused on a specific cell type, and the results may not be generalizable to other cell types or tissues. We highlight these limitations and experimental constraints so that the reader would be better informed to assess the scope and implications of this study.

## 4. Conclusions

The findings of this in vitro study suggest that nanoscale topographies with a comb-like pattern were formed on Ti surfaces optimized through etching with an eco-friendly solution. Additional etching with the etching solution caused the formation of nano/micro hierarchical structures, increased surface roughness, and enhanced hydrophilicity. Enhanced osteoblast adhesion and proliferation were observed on the modified surfaces. The eco-friendly etching method has the potential to enhance the biological properties of Ti implant surfaces and thereby improve dental implant performance. Therefore, the null hypothesis was rejected. Although additional etching of the SLA surface appears to be a promising approach, further research is required to comprehensively assess its merits.

## Figures and Tables

**Table 1:** Experimental groups of specimens considered in this study.

Group (*n* = 10)	Surface Treatment
M	No surface treatment
ModM	No surface treatment + eco-friendly solution ^(a)^ etching
SL	Alumina sandblasted
ModSL	Alumina sandblasted + eco-friendly solution etching
SLA	Alumina sandblasted + acid-etching
ModSLA	Alumina sandblasted + acid-etching + eco-friendly solution etching

^(a)^ 30 wt% H_2_O_2_/5 wt% NaHCO_3_ solution.

**Table 2:** Binding energy and atomic concentration (at%) for various surface modification treatments.

Element	Machined				Sandblasted				SLA			
	M		ModM		SL		ModSL		SLA		ModSLA	
	at%	BE	at%	BE	at%	BE	at%	BE	at%	BE	at%	BE
Ti 2p	5.2	459.0	19.6	458.6	16.3	458.1	17.7	458.1	14.0	458.2	21.0	458.1
O 1s	24.7	530.8	46.2	531.7	56.0	530.3	54.2	530.7	44.0	531.8	47.5	531.4
C 1s	69.9	284.9	34.1	285.3	27.5	284.8	27.9	285.0	41.9	285.2	31.3	285.0

BE: Binding Energy.

## Data Availability

Not applicable.
